# A few prolific liars in Japan: Replication and the effects of Dark Triad personality traits

**DOI:** 10.1371/journal.pone.0249815

**Published:** 2021-04-15

**Authors:** Yasuhiro Daiku, Kim B. Serota, Timothy R. Levine

**Affiliations:** 1 Graduate School of Human Sciences, Osaka University, Suita, Osaka, Japan; 2 Department of Management and Marketing, Oakland University, Rochester, Michigan, United States of America; 3 Department of Communication Studies, University of Alabama at Birmingham, Birmingham, Alabama, United States of America; Univeristy of Padova, ITALY

## Abstract

Truth-Default Theory (TDT) predicts that across countries and cultures, a few people tell most of the lies, while a majority of people lie less frequently than average. This prediction, referred to as “a few prolific liars,” is tested in Japan. The study further investigated the extent to which the Dark Triad personality traits predict the frequency of lying. University students (*N* = 305) reported how many times they lied in the past 24 hours and answered personality questions. Results indicate that the few prolific liars pattern is evident in Japan thereby advancing TDT. Results also show that Japanese frequent liars tend to have Dark Triad personality traits, but the nature of the findings may be unique to Japan. Results of the generalized linear model suggest that the Dark Triad components of Machiavellianism and psychopathy exacerbate lying behavior by reducing the guilt associated with lying. However, narcissism encourages guilt and therefore inhibits lying behavior with both direct and indirect effects. These narcissism findings appear to contradict prior studies but stem from use of a more appropriate statistical analysis or the Japanese context.

## Introduction

Most people lie at least once in a while, but how often people lie is frequently misunderstood. For example, DePaulo et al. [1] used a daily diary method to investigate both the nature and number of lies people tell. They found that college students told 1.96 lies and non-students told 0.97 lies on average per day. Murai [2] replicated their findings in Japan, obtaining very similar results. He asked undergraduate and graduate university students to record their lies for a full week and found that male students told an average of 1.57 lies per day and that female students told 1.96 lies. According to these averages, lying seems to be a ubiquitous, everyday phenomenon, and this is just how these findings are often interpreted in the literature [3].

Truth-Default Theory [3, 4], however, posits that the average is misleading in this case because the distribution of lying in human populations is heavily positively skewed. A few prolific liars cause the mean to depart from the median and mode. According to TDT, most people lie infrequently and most lies are told by a few prolific liars, and this pattern is predicted to be universal across human cultures. Simply put, when it comes to how often people lie, most people are not average.

TDT is a pan-cultural theory. TDT’s core presumptions concern human nature and are not tied to any particular culture, religion, government, or social structure. According to TDT, the prevalence and distribution of lying in communication are critical considerations because prevalence rates have implications for the utility of the truth-default. Truth-default is the idea that people passively and uncritically accept the content of incoming messages as honest and truthful unless some trigger event prompts suspicion [3–5]. The truth-default is functional and adaptive in a social world where deception is infrequent, but it is disadvantageous in environments and circumstances where deception is prevalent. Thus, the truth-default will only evolve in social environments where deception has a low base-rate [6]. It follows that investigating lie prevalence in various countries and cultures is theoretically important for advancing TDT because all the propositions and modules, including the few prolific liar pattern, are specified to be pan-cultural.

Finding infrequent lying is important to TDT because humans tend to believe others. Most often people are in a truth-default state in which they passively believe others and where thoughts about deception do not even come to mind. Even when suspicion is triggered, people still tend to be truth-biased. Believing others through the truth-default and truth-bias is advantageous if more people are honest (i.e., deception prevalence is low) [3, 6].

Initial support for the few prolific liar prediction from TDT was reported by Serota et al. [7] who surveyed a representative sample of 1,000 American adults, asking them to report how many times they have told a lie in the past 24 hours. As predicted, the distribution of the frequency of lies in a day was extremely skewed. The results indicated that 59.9% of participants reported no lies in the past 24 hours, while 7.9% reported six or more lies. One-half of all reported lies in the United States study were told by just 5.3% of the population. Seventy-five percent of respondents reported lying less frequently than the average (mean). The study concluded that “a few prolific liars” tell the majority of lies. The few prolific liars pattern of results has been subsequently observed in other countries such as the United Kingdom [8], Korea [9], the Netherlands [10, 11], and Israel [12]. These findings all challenge the classic view of “everyone lies every day” and support TDT.

The phenomenon of a few prolific liars appears to be broadly recurring and TDT’s predictions are pan-cultural [3]. However, research has been conducted in only a few countries and more data from other countries are needed to confirm its universality. Therefore, this study aimed to replicate the findings in Japan. Since the Murai study [2] did not report the data distribution we opted to collect new data in order to test the prolific liar hypothesis.

Extending beyond TDT predictions, we additionally investigated the personalities of prolific liars. TDT’s specification of a few prolific liars implies that there are individual differences in the proclivity to lie. If this is the case, then this individual variation may be explainable in terms of various personality traits and dimensions. Although numerous traits are associated with individual differences in communication, the Dark Triad psychological traits seem especially relevant due to their “dark” nature. The Dark Triad refers to socially aversive personality traits that include Machiavellianism, psychopathy, and narcissism [13]. Prior studies have indicated that the Dark Triad personality traits lead to people telling more lies [11, 14]. However, these studies may have not considered the distribution of lies during their analysis. For example, Halevy et al. [11] simply calculated the Spearman’s Rho correlation between Dark Triad and the frequency of lies. In our view, ignoring distribution characteristics sometimes leads to misconceptions; therefore, we tested this assumption using a more robust statistical model.

Besides Dark Triad, we wondered if other personality traits investigated by previous studies might mediate the effects of personality (the Dark Triad) on lie frequency. For example, Halevy et al. [11] indicated that prolific liars will be psychopathic people who presumably experience less guilt. Their argument appears reasonable, considering Dark Triad is theoretically characterized by lack of empathy [15] and empirically related toto feeling of guilt [16, 17]. Also, as Wright et al. [18, 19] pointed out, acceptance of lies and ability to detect lies are associated with ability to lie.

This study employs a generalized linear model (GLM), which is useful when a dependent variable is not normally distributed. In this study, we investigated the personality traits of a few prolific liars—including the Dark Triad—by using GLM while controlling other variables to replicate the long-tail phenomenon of a few prolific liars.

## Method

This study was approved by the Osaka University’s School of Human Sciences’ ethics committee (HB300-07). All participants provided written consent on the survey page.

The initial sample included 340 undergraduate students (187 males, 153 females, *M*_*age*_ = 19.61 ±3.33 years) at two Japanese universities. The first author called on university students to voluntarily answer the survey after a colleague’s class. Only students who agreed to answer participated in our study. Participants were excluded if they reported that they did not understand the instructions of this survey (5 participants), or marked a wrong number on the DQS (see below, 27 participants), or their first language was not Japanese (23 participants), or if they reported over 10,000 lies in the past 24 hours (1 participant). Several participants violated more than one of these criteria. After these exclusions, a total of 305 participants (174 males, 131 females, *M*_*age*_ = 19.39 ±3.31 years) were included in the analyses. This sample size provided statistical power of .94 to detect a zero-order correlation of *r* = .20 at *p* < .05.

The participants voluntarily answered our survey after their class. The survey included three sections: a lying frequency questionnaire, personality trait measures, and demographics. A confidence of response measure was also included.

The first section concerned lying frequency. These questions were a Japanese translated version of the one used by Serota et al. [7]. We asked participants how many times they had lied in the past 24 hours. Before reporting their lying frequency, they read the survey explanation provided and were then asked whether they understood the goal of the survey. After indicating their understanding, participants answered separately for lies to family members, friends, business contacts, acquaintances, and total strangers. For each type of receiver, they were also asked about lies in both face-to-face and mediated situations. If they answered 0 (no lies) in all boxes, they were asked the additional question: “When was the last time you did tell a lie to someone?” There were five answers from which to choose: “more than 24 hours ago but within the last two days,” “more than two days ago but within the last week,” “more than a week ago but within the last month,” “more than a month ago,” and “never.”

Linguistically, there are no big differences in meaning between the English “lie” and the Japanese 「うそ」. There may be a slight difference in nuance; the English “lie” sounds more negative, while Japanese 「うそ」 includes just joking. But, within this study, we used the same instruction as the original study [7], in which we explained that we are interested in all kinds of lies, and only those who understood the instruction participated the study. There should be no effects due to the difference in language.

After the lying frequency questions, subjects were asked to complete the personality traits section, including the Japanese version of the Dark Triad Dirty Dozen (DTDD) [20]. The DTDD is a validated scale for measuring the three components of the Dark Triad with four five-point items [21]. Participants also answered three items from Serota and Levine’s [8] UK study using a five-point scale: “Do you think there is such a thing as an acceptable lie?” indicating acceptance, “Do you ever feel guilty after telling a lie?” indicating feelings of guilt, and “Do you think you can tell when people are lying to you?” indicating perceived detection ability. We explored the potential effects of these three questions, although we did not have established hypotheses about these concepts.

To exclude random and biased responses, the personality traits questionnaire also included the directed questions scale (DQS) [22] and the Yanai et al. [23] lie scale. DQS is an item that asks participants to mark a specific option (e.g., “Mark four at this item”). Participants who do not read questions are likely to mark the wrong number. DQS was also set to preclude random responses. The Yanai et al. lie scale examines to what degree participants try to make themselves socially desirable. The lie scale included three items with five-point answers extracted from the Yanai et al.’s [23] 13-scale personality inventory (e.g., “I do not care about criticism from other people at all”). In the analysis, we initially aimed to use the lie scale as a control variable to suppress social desirability effects, but we excluded it from the analysis due to its extremely low reliability (α = .29).

At the end of the survey, participants provided demographic information and indicated confidence in the accuracy of their lie responses. Participants used a five-point scale to answer, “How accurate do you think the number of lies you reported was?” This question was added to confirm that the few prolific liars phenomenon is not merely an artifact of guessing as some participants might not remember how many times they had lied in the past 24 hours. The aim of this check was to investigate whether the phenomenon replicates even when we exclude unsure participants’ answers.

## Results

**Fig 1:**
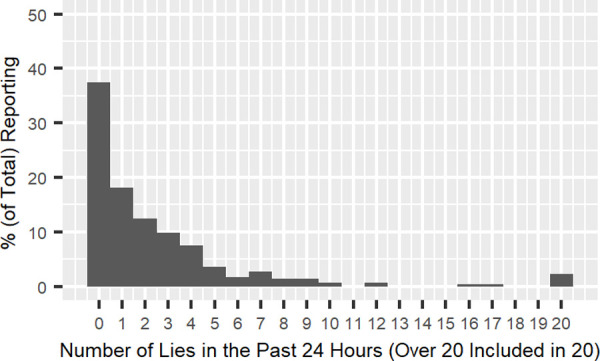
Total number of lies reported (all participants).

The number of lies in the past 24 hours were calculated and graphed (see Fig 1). Prior studies involving student samples generally report more lies on average than adult samples [1, 7]. Consistent with these higher rates, an average of 2.96 lies (*SD* = 9.50, *Median* = 1.00, *Range*: 0 to 150, *Mode* = 0) during the prior 24-hour period was reported. The mode was zero (37.4% of participants reported that they did not lie in the past 24 hours), and 51.5% reported that they told one to five lies. Only 11.1% reported six or more lies; but this group accounted for 59.3% of total reported lies (536 out of 904 lies).

Of the 114 participants who reported no lies in the past 24 hours, 36.0% answered “more than 24 hours ago but within the last two days,” 39.5% answered “more than two days ago but within the last week,” 14.9% answered “more than a week ago but within the last month,” 7.9% answered “more than a month ago.” Only 1.8% answered “never.” Overall, 96.4% of *total* study participants (including those who reported one or more lies in the past 24 hours) reported lying in the preceding month, consistent with monthly rates observed in United States studies [11].

**Fig 2:**
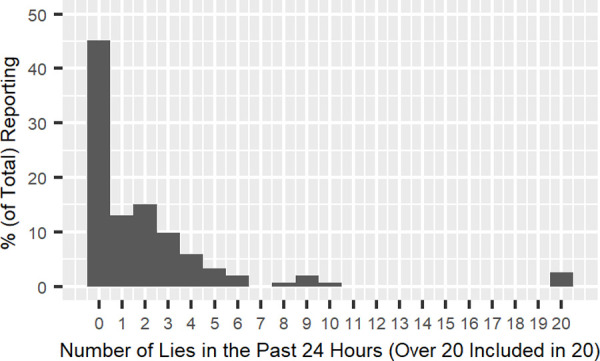
Total number of lies reported by high-confidence participants.

**Fig 3:**
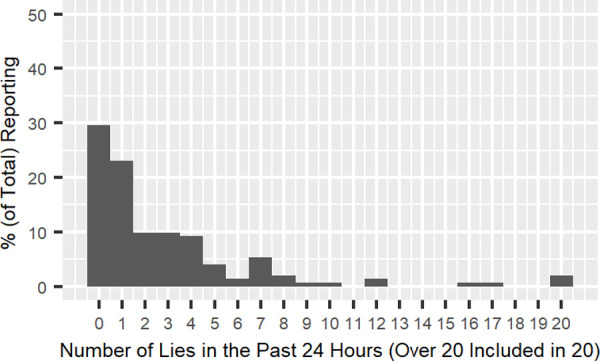
Total number of lies reported by low-confidence participants.

After excluding the 153 low-confidence participants (i.e., participants who marked one or two in the “confidence” question), the shape of the distribution was replicated (Fig 2). These participants told 2.14 lies on average (*SD* = 4.64, *Median* = 1.00, *Range*: 0 to 37, *Mode* = 0). Concerning the distribution, 45.4% of participants reported no lies in the past 24 hours, 47.4% reported one to five lies, and only 7.2% reported six or more lies, which accounted for 47.2% of the total reported lies (154 out of 326 lies). The distributions for the high-confidence and low-confidence participants were highly correlated (*r* (20) = .91, *p* < .001 and *r*^2^ = 0.82 for the raw frequencies; *r*^2^ = 0.89 for the power law trends fitted to the high- and low-confidence distributions; see Figs 2 and 3). Both forms of results show that the “few prolific liars” distribution observed elsewhere is also found in Japan. Since we observed very similar results regardless of perceived confidence, we used the complete data set in the next analysis.

To examine the personality traits of prolific liars, we calculated the zero-order Pearson’s correlations among the variables measured (above the diagonal in Table 1). The Dark Triad measures had minimally sufficient reliability (Machiavellianism: α = .76; psychopathy: α = .56; and narcissism: α = .77). The correlation analyses revealed that lying frequency was positively correlated with psychopathy (*r* [305] = .14, *p* = .012) and marginally correlated with Machiavellianism (*r* [305] = .10, *p* = .087). For narcissism, however, there was no significant correlation with lying frequency (*r* [305] = -.08, *p* = .168). In addition, guilt (*r* [305] = -.21, *p* < .001) was negatively correlated while perceived detection ability (*r* [305] = .10, *p* = .087) had a marginal correlation with lying frequency.

**Table 1:** Zero-order correlation between the variables.

	1	2	3	4	5	6	7	8	9	10
1. Sex (Male)		-.07	.10†	.16**	.17**	.15**	.01	.08	-.15**	.08
2. Age	-.04		.06	.00	-.04	-.01	.06	.10†	.03	-.02
3. Confidence	.10†	.04		-.03	-.07	-.10†	-.09	.09	.04	-.01
4. Machiavellianism	.16**	-.01	-.04		.49***	.32***	.17**	.24***	-.29***	.10†
5. Psychopathy	.16**	.00	-.09	.46***		.11†	.15**	.10†	-.27***	.14*
6. Narcissism	.16**	.05	-.10†	.31***	.11†		.10†	.04	.07	-.08
7. Acceptance	.03	.06	-.09	.23***	.15**	.14*		.08	-.18**	-.08
8. Ability	.07	.03	.08	.23***	.09	.03	.05		-.05	.10†
9. Guilt	-.16**	-.03	.04	-.28***	-.23***	.06	-.20***	-.02		-.21***
10. Lying. Freq.	.04	-.12*	-.18**	.21***	.14*	.12*	.11*	.09	-.19**	

Note: Entries above the diagonal are Pearson’s correlation coefficients, and below the diagonal are Spearman’s rank correlation coefficients.

† *p* < .10,

* *p* < .05,

**p < .01,

*** *p* < .001.

Since the distribution of lying frequency was very skewed, we also calculated Spearman’s rank correlations (below the diagonal in Table 1). The Spearman’s correlations of lying frequency with other variables were different from the Pearson’s. For example, in the Spearman’s correlations, Machiavellianism (*r* [305] = .21, *p* < .001) and narcissism (*r* [305] = .12, *p* = .043) as well as psychopathy (*r* [305] = .14, *p* = .014) was positively correlated with lying frequency. In addition, perceived detection ability did not have a significant correlation with lying frequency (*r* [305] = .09, *p* = .105), while guilt (*r* [305] = -.19, *p* = .001) and acceptance (*r* [305] = .11, *p* = .045) had significant correlations.

These difference between Pearson and Spearman’s correlations suggested that we need a more sophisticated analysis that can accurately model the distribution of lying frequency. Therefore, we conducted a hierarchical regression using the Generalized Linear Model, regarding lying frequency as count data. Since a Poisson regression caused an overdispersion due to zero-inflation, we used a negative binomial regression. We placed control variables in Step 1, Machiavellianism, psychopathy, and narcissism in Step 2, acceptance in Step 3, ability in Step 4, and guilt in Step 5 (Table 2). Table 2 also shows the means and standard deviations. Based on the net increase of Cox and Snell’s pseudo *R*^*2*^, the composite Dark Triad measure and guilt had greater influences compared with other variables. Moreover, only narcissism was significant in Step 5, although all aspects of the Dark Triad had significant effects in Step 2. This suggests that guilt mediated the effects of the Dark Triad, but narcissism by itself may have direct effects when controlling for other variables.

**Table 2:** Results of the hierarchical negative binomial regression.

	Step1	Step2	Step3	Step4	Step5	*Mean* (*SD*)
Intercept	1.56*	1.63*	2.30**	1.78*	3.43***	
Sex (Male)	0.61***	0.40*	0.39*	0.37*	0.18	
Age	-0.03	-0.04	-0.03	-0.04	-0.04	19.39 (3.31)
Confidence	-0.08	-0.18*	-0.20**	-0.20**	-0.19**	2.84 (1.20)
Machiavellianism		0.28*	0.31*	0.23†	0.09	2.78 (0.87)
Psychopathy		0.29*	0.25†	0.27*	0.14	2.77 (0.77)
Narcissism		-0.37***	-0.35**	-0.32**	-0.21*	3.43 (0.85)
Acceptance			-0.17	-0.15	-0.15	4.41 (0.72)
Ability				0.19*	0.17*	3.09 (1.01)
Guilt					-0.33***	3.28 (1.12)
*θ*	.47	.55	.55	.57	.62	
Cox & Snell *R*^*2*^	.04	.14	.15	.16	.21	

Note: *θ* is the shape parameter of the negative binomial distribution.

† *p* < .10,

* *p* < .05,

**p < .01,

*** *p* < .001.

**Fig 4:**
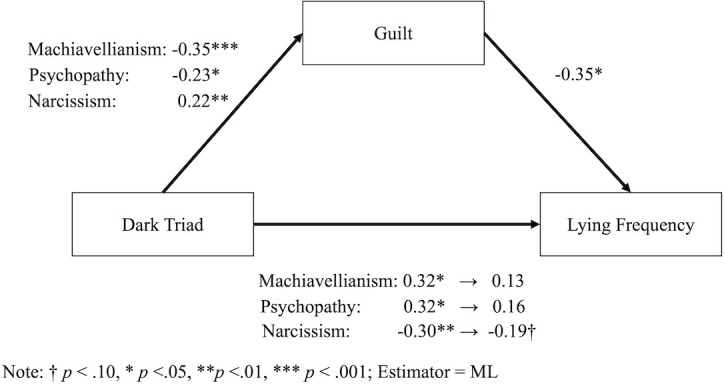
Models of the generalized SEM for mediation effects.

To investigate the mediation effects in more detail, we conducted a generalized SEM (Fig 4) by using Mplus (Version 7) [24]. This analysis assumed that the number of lies was subject to a negative binomial distribution. We first estimated the direct effect of the Dark Triad (Machiavellianism, psychopathy, and narcissism simultaneously) on lying frequency. Then, we added guilt as the mediating variable while retaining direct paths. Finally, we estimated indirect effects and conducted Sobel tests [25] (see Table 3). The results showed that guilt mediated the effect of the Dark Triad. Interestingly, narcissism had a positive effect on guilt, while Machiavellianism and psychopathy had negative effects. Moreover, the direct effect of narcissism remained (although marginally) while the direct effects of Machiavellianism and psychopathy vanished.

**Table 3:** Results of indirect effects in the generalized SEM for mediation effects.

	*Est*.	*SE*	95%*CI*	*Z*
Machiavellianism × Guilt	0.12	0.04	0.04 − 0.20	2.98**
Psychopathy × Guilt	0.08	0.04	0.01 − 0.15	2.20*
Narcissism × Guilt	0.08	0.03	-0.14 − -0.02	-2.46*

Note:

† *p* < .10,

* *p* < .05,

**p < .01,

*** *p* < .001.

## Discussion

The purpose of this study was to test the few prolific liars predictions in Japan and to examine these prolific liars’ personality traits. Consistent with TDT predictions, the results documented the existence of the few prolific liars pattern in the current sample of Japanese students. Moreover, the results demonstrate that people high in Machiavellianism and psychopathy reported more lying, mediated by lowering guilt, while people high in narcissism reported less lying through both direct and indirect paths. Although we cannot fully establish the causal relationships with only this study, the results suggest that people high in Machiavellianism or psychopathy may be inclined to tell more lies due to *reduced* feelings of guilt and that people high in narcissism may tell fewer lies due to *increased* guilt. The reverse causal order alternative is that the act of lying reduces guilt causing Machiavellianism scores to increase. While it is possible that people who lie frequently come to experience less guilt over time, and as a consequence, rate themselves as higher on Machiavellianism and psychopathy, this seems less plausible than personality being the antecedent.

Consistent with prior studies, the distribution of self-reported lies is extremely skewed, indicating the existence of a few prolific liars in our sample. The average lying frequency was similar to that reported by prior studies, such as DePaulo et al. [1], Murai [2], and Serota and Levine [8]. Most participants reported five or fewer lies in the past 24 hours and only a few people reported six or more lies. Importantly, prior results demonstrate that the few prolific liar phenomenon is not an artifact of the self-reporting methodology. Halevy et al. [11] showed that the self-reported number of lies correlates with behavioral indices of dishonesty in a laboratory and in our data, eliminating low-confidence participants does not change the overall finding. Therefore, the self-reported results appear to represent a reliable index and the universality of the “few prolific liars” module of TDT.

TDT seeks to provide a pan-cultural account of human deceptive communication. Because TDT predictions are not culturally bound, it is critical to test TDT in a variety of cultures. Only by testing TDT in various countries can the robust nature of TDT’s predictions be ascertained. Although TDT studies have previously been conducted in North America, South America, Europe, Asia, and the Middle East, this research is the first to test TDT in Japan. The current findings add to the cultural span of TDT by replicating effects documented elsewhere.

Investigating the personality traits of the prolific liars using GLM yielded a more complex outcome than prior results. These results showed that Machiavellianism and psychopathy are associated with more lying, similar to prior studies [11, 14]. This suggests the two effects are robust enough to endure more rigorous statistical analysis. In addition, this study revealed that the effects are mediated by reduced feeling of guilt. Those high on Machiavellianism and psychopathy are thought to have lower guilt than ordinal people do, and this lower inhibition contributes to telling more lies.

These results, that the few prolific liars are Machiavellian and psychopathic people, may shed light on the fundamental question, “why is the distribution so skewed?” from an evolutionary perspective. Previous research found that people who have Dark Triad personality traits take the fast life strategy characterized by short-term mating, selfishness, and other antisocial manifestations [15, 26] and that they account for only a small part of the entire population [27]. Considering these findings, one possible explanation for the skewed distribution of lying is that the few prolific liars are people who adopted the fast life strategy. In modern society, the traits are seen as undesirable because most people do not adopt this strategy [28] but prolific lying may help those who adopt the fast life strategy to survive and reproduce. This evolutionary system may be the reason why we see the few prolific liars across cultures. This hypothesis is speculative but warrants further investigation.

However, somewhat surprisingly, narcissism had a negative effect on the frequency of lying. That is, results show people high in narcissism tell fewer lies. This result is contradictory to prior studies, which may result from the choice of statistical analyses. Jonason et al. [14] calculated the correlation coefficients and partial regression coefficients, finding a slightly positive correlation between narcissism and the number of lies. Similarly, Zvi and Elaad [12] found a positive relationship between narcissism and lying behavior. However, without accounting for the extremely skewed distribution of lie frequency, calculating Pearson correlations may yield misleading results, especially Type I errors [29]. As this and prior studies [7, 8, 11] indicated, approximately 40–60% of people asked about lying frequency report no lies during any specific 24-hour period. Therefore, the distribution for lying frequency will be positively skewed and substantial (Skewness > 1.0 is considered substantial; for the Japan data Skewness = 12.67, SE of Skewness = 0.14). This inclination is not only an extreme deviation from the assumption of normality, it is wholly unsuitable for calculating Pearson’s correlations, which assume linear relationships between two variables. In addition, just a few prolific liars might exorbitantly increase the correlation, as Pearson’s correlation is very sensitive to outliers. For these reasons, Pearson’s correlations with lie frequency may be unreliable when the skewed distribution is considered. Spearman’s rank correlation suppresses the effect of outliers.

Moreover, we found the negative effect for narcissism (i.e., narcissists tell fewer lies) when controlling Machiavellianism and psychopathy. While the zero-order correlations of narcissism include the effects of Machiavellianism and psychopathy, the result of the negative binomial regression partials out the effects of them when assessing the effect of narcissism. Thus, it may be safe to say that the negative coefficient of narcissism is the pure effect of narcissism on lying frequency. This may be the reason why we had the negative coefficient while we had a positive correlation between lying frequency and narcissism in Spearman’s rank correlation.

This negative effect of narcissism on lying is interpretable from three perspectives. The first is narcissism’s relative brightness. Narcissism is considered the least dark trait among the three [30]. Narcissism has weaker relationships with anti-social behavior [15, 31, 32] and the ability to lie [33] than do either Machiavellianism or psychopathy. Considering these findings, perhaps it is not so surprising that narcissism had a different effect from Machiavellianism and psychopathy in our study. Narcissism is characterized by entitlement, superiority, and dominance [14]. The narcissist’s priority is keeping self-image positive, and frequent lying may hurt self-image. If so, it may be a reason why those higher on narcissism tell fewer lies.

The second consideration is lying types. Our study did not classify lying types, so all kinds of lies are included in the analysis. Narcissists are thought to tell lies mostly about themselves to make a good impression on others. In fact, Jonason et al. [14] revealed that narcissism had its strongest relationship with the number of self-gain lies. Future research might benefit by classifying lie types as well as motives to lie.

The third possibility is cultural differences. Narcissism scores may differ across countries. Foster et al. [34] found that narcissism was higher in an individualistic culture than in a collectivistic culture; the United States, especially, produced the highest levels of reported narcissism. According to their study, Japan’s narcissism is predicted to be lower than that of the United States. Moreover, Japan is thought to have a shame culture rather than a guilt culture [35], suggesting that in Japan, social behavior might be determined by feelings of shame rather than guilt. Replicating the current study in a western country could facilitate a comparative cultural analysis.

Further research on the subtypes of narcissism also might be useful for interpreting this result. Narcissism can be divided into vulnerable narcissism—associated with introversion, defensiveness, anxiety and vulnerability to life’s traumas—and grandiose narcissism—associated with extraversion, self-assurance, exhibitionism, and aggression [36]. Previous research has revealed that grandiose narcissism is more strongly related to unethical behaviors than vulnerable narcissism [16]. The Dark Triad Dirty Dozen, which we used in the current study, does not measure the two types separately. Consequently, there is a possibility that the DTDD is primarily measuring vulnerable narcissism and that this form of narcissism, which is associated with a positive self-image, is more likely to inhibit lying.

The current study has three limitations to consider. First, our analysis did not control for the frequency of social interaction. The Dark Triad personality traits are positively correlated with extraversion among the Big Five personality traits [13]. Thus, an alternative explanation for high lie frequency could be that prolific liars have more social interactions in a day rather than having an anti-social personality. However, studies that have controlled for frequency of interaction [1, 37] found prolific liars even with a known rate of interaction. Future research may resolve this point by controlling for interaction rate.

Second, the results of this study are based solely on lies reported by college students. To improve the generalizability of the results, a study obtaining lie reports from a broader sample could be conducted. Fortunately, research in other countries is informative about how student samples are similar and different from more broadly representative samples. Research has documented the few prolific liars pattern (i.e., positive skew) in studies of both students and adult samples [7, 8, 10]. The primary difference is that students tend to tell more lies on average. It is reasonable to expect that we would find a similarly skewed distribution among Japanese adults even though they may tell fewer lies, overall.

Third, the measurement of the Dark Triad used in this study may be insufficient. The Japanese version of the DTDD has differences from the original English version (e.g., lower reliability of psychopathy). The differences are most evident in Machiavellianism and psychopathy, but due to the strict translation procedures they are not substantial. It appears unlikely that the divergence for narcissism may have resulted from a translation problem.

Future research might examine other TDT propositions in Japan and other countries in Asia. Truth-bias has been documented in Korea [6] and Murai [2] found that Japanese participants reported (knowingly) receiving far few lies each day than they told. Both prior findings are consistent with TDT’s applicability in Asian countries. Future research might provide a more direct test of the truth-default using the method developed by Clare and Levine [5] thus investigating if thoughts of deception come to mind unprompted. Given known cultural differences (e.g., collectivism versus individualism; power distance), TDT’s predications regarding pan-cultural deception motives and the projected motive model also need to be tested across Asia.

Overall, this research clearly indicates the existence of a few prolific liars in a student sample in Japan. As observed in other parts of the world, most Japanese people tell few or no lies on a given day and a small number of people, prolific liars, tell the majority of lies. Additionally, the study found that lying frequency increased with higher Machiavellianism and psychopathy scores, and that these factors are mediated by feelings of guilt. Documenting the mediating effects of guilt expands our knowledge about lying and its prediction. This mediating effect suggests that people with certain personality traits such as Machiavellianism may feel less guilty about lying and consequently have fewer inhibitions about lying. Practically, it may be effective to activate people’s feelings of guilt to suppress lying in real world. We further observed an unexpected effect of narcissism, which inhibited lying frequency. How narcissism affects lying should be investigated further.

## Supporting information

S1 File (CSV)

S2 File (DOCX)
