# Supplementary material for: A few prolific liars in Japan: Replication and the effects of Dark Triad personality traits
Source: PLoS One. 2021 Apr 15;16(4):e0249815. doi: 10.1371/journal.pone.0249815 (PMC8049473; doi:10.1371/journal.pone.0249815)
Supplement: S2 File — (DOCX) [file pone.0249815.s002.docx]

**How many times have you lied? – Questionnaire Wording**

Initially used for the research reported in:

Serota, K. B., Levine, T. R., & Boster, F. J. (2010). The Prevalence of Lying in America: Three Studies of Self‐Reported Lies. *Human Communication Research*, *36*(1), 2-25.

This is the basic question used for the Serota et al. (2010) US national survey:

We are interested in truth and lies in people’s everyday communication.

Most people think a lie occurs any time you intentionally try to mislead someone. Some lies are big while others are small; some are completely false statements and others are truths with a few essential details made up or left out. Some lies are obvious, and some are very subtle. Some lies are told for a good reason. Some lies are selfish, other lies protect others. We are interested in all these different types of lies. To help us understand lying, we are asking many people to tell us how often they lie.

Think about where you were and what you were doing during the past 24 hours, from this time yesterday until right now. Listed below are the kinds of people you might have lied to and how you might have talked to them, either face-to-face or some other way such as in writing or by phone or over the internet. In each of the boxes below, please write in the **number of times you have lied** in this type of situation. If you have not told any lies of a particular type, write in “0”.

In the past 24 hours, how many times have you lied?


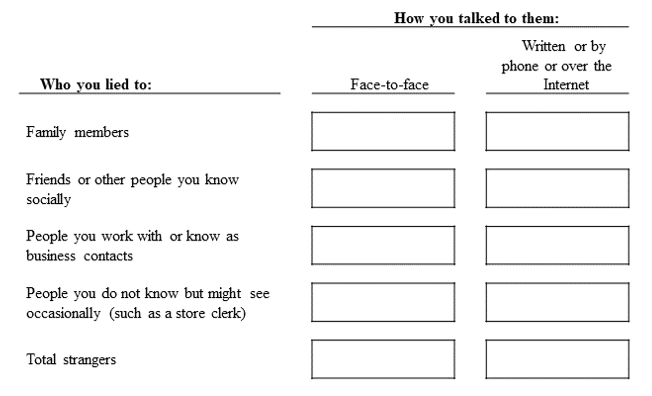


The following question was not part of the national survey but was asked in Study 3 (student sample) and has been asked of subjects in subsequent studies in order to establish less frequent lying behavior:

[If you wrote “0” in all 10 boxes of the previous question, please answer the following:] You indicated that you did *not* tell any lies in the past 24 hours. When was the last time you did tell a lie to someone? [check one box]

🞏 More than 24 hours ago but within the last two days

🞏 More than two days ago but within the last week

🞏 More than a week ago but within the last month

🞏 More than a month ago

🞏 Never
